# Atomistic Insight Into the Host–Guest Interaction of a Photoresponsive Metal–Organic Framework

**DOI:** 10.1002/chem.201905139

**Published:** 2020-01-21

**Authors:** Elena Kolodzeiski, Saeed Amirjalayer

**Affiliations:** ^1^ Physikalisches Institut Westfälische Wilhelms-Universität Münster Willhelm-Klemm-Strasse 10 48149 Münster Germany; ^2^ Center for Nanotechnology (CeNTech) and Center for Multiscale Theory and Computation (CMTC) Heisenbergstrasse 11 48149 Münster Germany

**Keywords:** atomistic simulations, host–guest interactions, metal–organic frameworks, molecular switches, photoresponsive materials

## Abstract

Photoresponsive functional materials have gained increasing attention due to their externally tunable properties. Molecular switches embedded in these materials enable the control of phenomena at the atomic level by light. Metal–organic frameworks (MOFs) provide a versatile platform to immobilize these photoresponsive units within defined molecular environments to optimize the intended functionality. For the application of these photoresponsive MOFs (pho‐MOFs), it is crucial to understand the influence of the switching state on the host–guest interaction. Therefore, we present a detailed insight into the impact of molecular switching on the intermolecular interactions. By performing atomistic simulations, we revealed that due to different interactions of the guest molecules with the two isomeric states of an azobenzene‐functionalized MOF, both the adsorption sites and the orientation of the molecules within the pores are modulated. By shedding light on the host–guest interaction, our study highlights the unique potential of pho‐MOFs to tailor molecular interaction by light.

## Graphical abstract

**Light on interaction**: Photoresponsive molecular switches embedded within metal–organic frameworks enable the modification of both the adsorption sites and the orientation of guest molecules. Atomistic simulations predict the high potential of these photoresponsive materials to change intermolecular interactions (see figure).

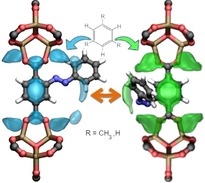

Stimuli‐responsive molecular switches enable the transfer of external stimuli such as light into well‐defined molecular motion. Embedded in precise molecular environments, these stimuli‐responsive molecular units perform a variety of essential functions in biological systems.^1^ Understanding the externally induced process and in particular the influence on the molecular surrounding opens the door for designing new functional materials with tunable properties.^2^ The potential of corresponding functional materials has been shown and the field of applications ranges from on‐command drug delivery over molecular sensing and catalysis to gas‐storage.^3^ Alike to the biological systems, in all these applications, intermolecular interactions and dynamic processes at the nanoscale modified by the molecular switching unit play a fundamental role for the resulting functionality. Thus, for the development of advanced photoresponsive functional materials, a detailed insight into structural and dynamic phenomena influenced by the molecular switch is crucial to tune the functionality.

In this context, azobenzene has been one of the most employed molecular building units.^4^ By photoexcitation, azobenzene can be interconverted between two states (*cis*/*trans*), which differ distinctively in their physical and chemical properties.^5^ To transmit the local photoinduced structural change into long‐range outcome, the molecular embedding of the azobenzene unit is fundamental. It is important to distinguish the case of high molecular confinement, in which the interconversion process might directly be influenced by the surrounding,^6^ and the case of integrating in open molecular structures, where interferences with the switching dynamics can be minimized.

Soft porous materials such as metal–organic frameworks (MOFs) enable, due to their tool‐kit assembly,^7^ the construction of highly ordered and tunable 3D structures with high porosity, providing a versatile approach to immobilize photoswitches. However, despite the increasing numbers of synthesized photoresponsive MOFs (pho‐MOFs), which exhibit promising properties,^8^ a thorough understanding of the influence of the photoresponsive units on the host–guest interaction is still limited and only a few numbers of theoretical studies have been reported.^9^ Herein, we reveal the impact of the different states of the photoswitch on the host–guest interaction. In particular, we predict how the position and orientation of guest molecules within the pho‐MOF are modulated, which is fundamental for applications such as catalysis. Therefore, we investigated a functionalized MOF‐5, which has a cubic network topology7c, 10 and extended the terephthalic acid linkers connecting the Zn_4_O tetrahedra corners in MOF‐5 by azobenzene derivatives (azo‐MOF‐5, Figure 1 a) similar to reported pho‐MOFs.9a, 11 By studying benzene derivatives as prototypical guest molecules in two azo‐MOF‐5 states (*all‐trans/all‐cis*), we show further that the effect of the switching unit depends on even minor structural modifications and differences of the guest molecules, opening the path to tailor multicomponent systems by light.

**Figure 1:**
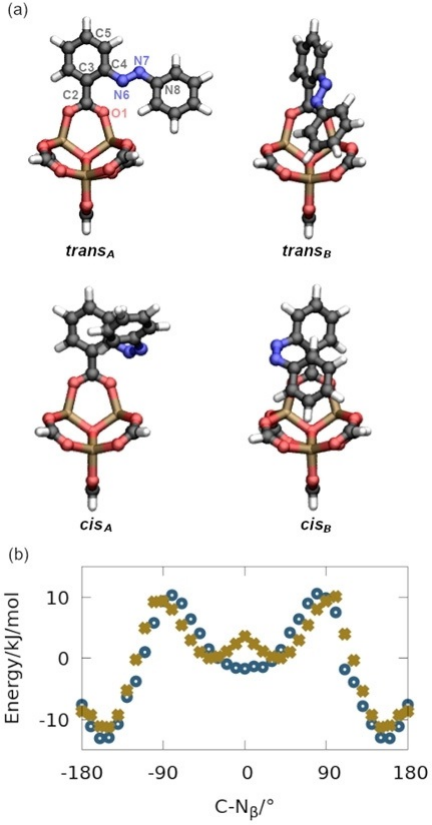
a) Ball and stick model of the optimized *trans* (***trans***_***A***_, ***trans***_***B***_) and *cis* isomers (***cis***_***A***_, ***cis***_***B***_) of the nonperiodic reference system (carbon: black, hydrogen; white, oxygen: red, zinc: brown). b) Energy profile along the rotation of C−N_β_ connecting ***trans***_***A***_ and ***trans***_***B***_ calculated with the force field (blue circles) and at the B3LYP+GD3 level (green crosses). The local minimum around 0° corresponds to ***trans***_***A***_ and the absolute minimum around ±154° corresponds to ***trans***_***B***_. The energy is given with respect to ***trans***_***A***_ calculated on DFT level.

The high potential of atomistic simulations applying classical force fields, which allow to cover large time and length scales, to advance the understanding of MOF‐based materials has been discussed extensively.^12^ Since for an accurate description of the host–guest system the employed force field is crucial, we extended our previously developed ab‐initio parametrized force field for MOF‐5 (see Figure S13 and Tables S1–S6, Supporting Information).^13^ Our main goal is to cover relevant parts of the ground‐state energy landscape to accurately describe both switching states within one force field (Scheme 1), which is different to other studies, in which a force field was developed to describe the switching process.^14^ In line with our reported approach and utilizing a genetic algorithm,^15^ we parametrized the force field based on quantum mechanical (QM) reference data calculated for a nonperiodic model of azo‐MOF‐5, consisting of one inorganic cluster with a single azobenzene unit (Figure 1 a). We used the structural and dynamic properties (geometry and hessian matrix) of one *trans* isomer (***trans***_***A***_) together with the energy differences (with respect to ***trans***_***A***_) of in total four stationary points of both *cis* and *trans* isomers, which differ mainly by the torsion around C5‐C4‐N6‐N7 (C‐N_β_, Figure 1 a), and of ***trans***_***A***_ rotated by 90° around C2−C3 (Figure S1, Supporting Information). The derived classical model is able to describe both the structural and dynamic properties of the four isomers of the model in excellent agreement with the QM data (Figures S2–S7, Supporting Information). Going beyond the stationary points, we validated further our force field by calculating the energy profile connecting the ***trans***_***A/B***_ conformers. Although the intermediate structures were not included in the fitting procedure, the energy data calculated by our parametrized forced are well in line with corresponding QM calculations (Figure 1 b). Due to small structural differences of the optimized ***trans***_***A***_ state (mainly the C−N_ß_: 5° (force field) and 36° (B3LYP+GD3), respectively, see also Figures S2–S6, Supporting Information), a small deviation around 0° is found. However, since we are interested in calculating the host–guest interactions at 300 K, the low energy barrier between the two configurations around 0° of 4.3 kJ mol^−1^ at the DFT level can be readily be overcome during the molecular dynamics simulations and thus only play a minor role for the further analysis.

**Scheme 1:**
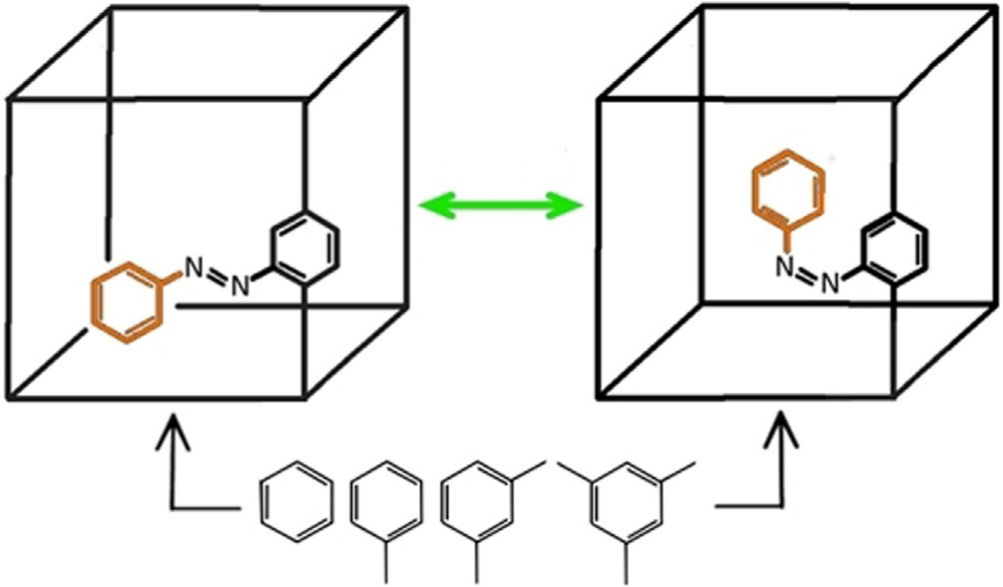
Schematic representation of azo‐MOF‐5 and investigated guest molecules. The immobilized benzene molecules (IBMs) are marked in orange.

The influence of the rotational degrees of freedom of the flexible units on the adsorption sites in MOFs has previously been investigated in different studies.^16^ In the current work we want to go a step further by analyzing the contribution of an externally tunable linker on the adsorption sites in order to understand the correlation between the photoresponsive linkers and the guest molecules. Therefore, we applied the validated force field to perform Molecular Dynamic (MD) simulations of the unloaded and loaded azo‐MOF‐5. Interestingly, already the analysis of the MD trajectories of the empty azo‐MOF‐5 indicates an influence of the linker conformation on the guest molecules (Figure 1 a). Before going into the analysis, it should be pointed out that due to the orientation of the organic linker two cell types (A and B) are present in MOF‐5‐type systems.10b

Keeping this in mind, the free phenyl units of the azobenzene linkers in *all‐trans* azo‐MOF‐5 point predominately into the B‐cells. In contrast, the free phenyl rings in *all‐cis* azo‐MOF‐5 can be considered as immobilized benzene molecules (IBM, Scheme 1) since they occupy the so‐called α‐pockets in the corners of the A‐cells (Figure 2 a), which have been previously identified as the primary adsorption sites for guest molecules in MOF‐5 related systems.15d, 17 Since the two switching states alter the α‐pocket, a modulation of the host–guest interaction is expected.

**Figure 2:**
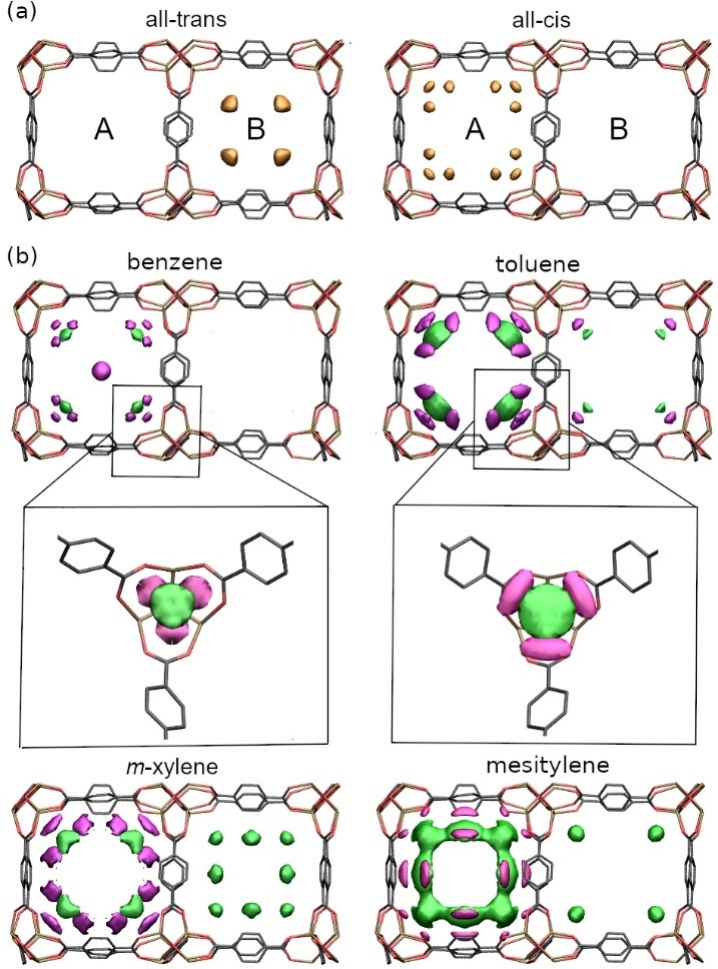
a) Probability distribution of the IBMs in *all‐trans* azo‐MOF‐5 (left) and *all‐cis* azo‐MOF‐5 (right). b) Difference of the probability distributions (*ρ_trans_*−*ρ_cis_*) between *all‐trans* and *all‐cis* for the benzene derivatives. (green denotes positive and violet negative values). For benzene and toluene, a zoom‐in of the corners of the A‐cell is depicted. The framework is shown as a guide to the eye and the same color coding as in Figure 1 a is used. The azobenzene units are omitted for clarity.

Our atomistic simulations of the benzene loaded azo‐MOF‐5 indeed confirm this hypothesis, by revealing a change of the interaction of the benzene molecules with the primary adsorption site. In the *all‐cis* azo‐MOF‐5 the benzene molecules interact with both the inorganic unit and the IBMs. As a result, the primary adsorption site in the corners of the A‐cell features a threefold partitioning (Figure 2 b, Figure S9, Supporting Information). In addition, the γ‐site, which is located in the center of the A‐cell and has only been reported at high guest molecule loading,15d, 18 is already energetically preferred at the considered lower guest molecule loading due to the interaction with the IBMs. In the *all‐trans* azo‐MOF‐5 the interaction with the α‐pockets is significantly less influenced by the IBMs and the host–guest interaction is comparable with the loaded nonresponsive framework.15d, 17, 19 Hence, the embedded molecular switches influence the primary interaction within the pores and, in addition, the host‐guest interaction can be switched between a low‐density and a high‐density configuration. Considering the toluene, *m*‐xylene and mesitylene (Figure 2 b, Figures S10–S12, Supporting Information), the modulation of the primary adsorption site becomes even more pronounced. Due to the increasing size of the guest molecules, the interaction with the inorganic unit decreases and the attractive interaction with the IBMs increases leading to a further shift of the guest molecules away from the corner towards the linkers in *all‐cis* azo‐MOF‐5. (Figure 2 b). At the same time, an increased population of the B‐cell and not of the γ‐site by the benzene derivative is observed in *all‐trans* azo‐MOF‐5 due the IBMs preferentially located in the B‐cell (Figure 2 b, toluene, *m*‐xylene).

In view of MOFs as solid solvents, the predicted modulation of host–guest interactions by molecular switches, highlights how molecular interaction and packing can externally be tuned by light within pho‐MOFs. However, for applications such as catalysis, not only the position but also the relative orientation of the guest molecules plays a crucial role. To map out if the different states of the embedded molecular switches affect the orientation of the guest molecules within the pores, we further evaluated our atomistic simulations and indeed observe an impact on the orientation. Since more than 85 % of the guest molecules are located in the A‐cell, we concentrated on analyzing the orientation of the guest molecules in this cell. The orientation of the molecules within the framework is examined by considering an angle *θ*, which describes the orientation between the surface normal of the aromatic ring of the guest molecules and a vector representing the diagonal of the A‐cell nearest to the guest molecule (Figure 3 a). For benzene, toluene and *m*‐xylene, no significant difference between *all‐cis* and *all‐trans* azo‐MOF‐5 is observed with respect to the orientation (Figure S8a–c, Supporting Information). In contrast, inspecting the probability distribution of *θ* for mesitylene, a dependency with respect to the switching state is evidenced (Figure S8d, Supporting Information). Despite the preferred orientation for the mesitylene molecules at *θ*=30–60°, a distinct change in the probability distribution is observed between *all‐cis* and *all‐trans* azo‐MOF‐5. In *all‐cis* azo‐MOF‐5 the probability of molecules with *θ*=60–90° increases by a factor of almost two, marking the influence of the two switching states on the orientation of the mesitylene within the pho‐MOF. To get a detailed picture of the host–guest interaction, we dissected the orientation of each molecule with respect to its center of mass. The corresponding probability distribution illustrates a clear correlation between the adsorption sites and the orientation (Figure 3 a, b). In *all‐cis* azo‐MOF‐5 the mesitylene molecules with *θ*=60–90° are predominantly located at the corners of the A‐cell but at a larger distance compared to the α‐pockets. In contrast to the *all‐trans* azo‐MOF‐5, the mesitylene molecules strongly interact with the IBMs in the *cis* configuration (Figure 3 d). As a result, three orientation‐dependent adsorption sites (ODASs) can be distinguished with respect to the interaction of the mesitylene (Figure 3 d): (i) primarily with the inorganic unit (*θ*=0–30°); (ii) with both the inorganic unit and the organic linker (*θ*=30–60°) and (iii) primarily with the IBMs (*θ*=60–90°). This assignment is supported by the RDFs between the mesitylene molecules and the IBMs, which provide information about the linker–guest interaction at each ODAS (Figure 3 c). Comparing the first peak of these RDFs, shows a shift to smaller values, which indicates an increasing interaction between the IBMs and the molecules for higher *θ* angles. This is in particular shown for the all‐*cis* MOF system. Hence, the ODAS with *θ*=75–90° (blue site in Figure 3) is clearly dominated by the interaction between the linker and the guest molecules. The correlation between the linker rotation and the adsorption sites is further highlighted by a structural analysis of the C−N_ß_ angle of the azobenzene linker, which is nearest to the guest molecule. From the MD simulations, two preferred positions can be identified for the loaded *all‐trans* case (C−N_ß_: 158° (A) and 39° (B)). Note that the deviation compared to the reference models (Figure 1) are due to the interaction of the linkers with the guest molecules and the MOF matrix itself. Remarkably, depending on the ODAS, the population of the A‐ and B‐state of the closest linker changes. In the *all‐trans* case the ration changes from 70:30 % for *θ*=0–30° to 30:70 % for *θ*=60–90°. In the all‐*cis* framework a similar correlation between linker rotation and guest molecules orientation is observed. Here, the population of the preferred C−N_ß_ angle of around 98° (C) and 56° (D), respectively, changes from 83:17 % for *θ*=0–30° to 75:25 % for *θ*=60–90°. The significantly different host–guest interactions for different ODAS depending on the photo‐switching state is further clarified by extracting the corresponding molecular configurations from our atomistic simulations (Figure 3 e).

**Figure 3:**
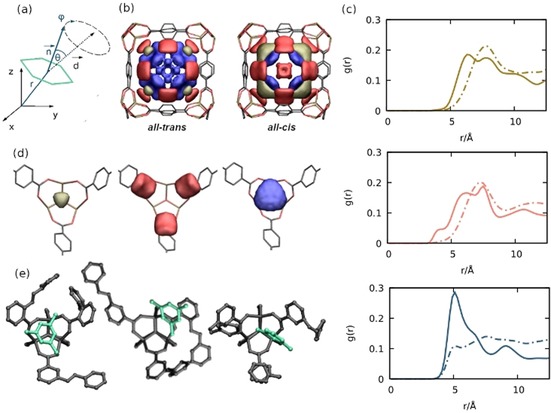
a) Schematic representation of the angle *θ* (*n̄*=normal vector of the guest molecule, *d̄*=diagonal vector of the A‐cell. b) ODAS topology (adsorption sites) with *θ* =0–30° (brown), 30–60° (red) and 75°–90° (blue). c) Radial distribution function between the mesitylene molecules and the center of mass of the IBM (for the brown, red, and blue areas in b). d) ODASs around the α‐pocket in ***all‐cis*** azo‐MOF‐5. e) Ball‐and stick model of representative structures of ODASs in azo‐MOF‐5.

In conclusion, we have investigated the influence of molecular switching units, incorporated within the matrix of MOFs on the host–guest interaction by atomistic simulation. A detailed molecular picture of the impact of the two switching states on the intermolecular interaction within the porous of these pho‐MOFs is presented by employing an ab‐initio parametrized force field. Experimentally so‐far not accessible, we predict how the photoresponsive building units modulate the preferred adsorption sites within the framework. Moreover, our study reveals the potential to alter even the orientation of the guest molecules by tailoring the host–guest interaction by light. At the same time our study shows that guest molecules with only small structural variations are affected differently by the embedded photoswitch. This highlights the potential of these photoresponsive functional materials to tailor systematically even multi‐component reactants in catalytic applications.

## Computational Details

The classical molecular mechanics calculations, including the Molecular Dynamic (MD) simulations, were performed using a locally modified version of the TINKER program package.^20^ Following our previous scheme we utilized a genetic algorithm (GA)15a–15c to parametrized our MM3 extended force field^13^, ^21^ based on DFT calculations. For the periodic calculations of the pho‐MOF, a unit cell consisting of eight Zn4O‐corners and 24 organic linkers was used. In order to generate the starting configurations and velocities for the microcanonical ensemble (NVE) MD simulations, 10 unloaded and loaded (6 guest molecules per unit cell) pho‐MOF systems (in both switching states (all‐*trans*/all‐*cis*) were equilibrated at 300 K for 0.5 ns using the Nose‐Hoover thermostat.^22^ The starting configurations for these NVT ensembles, were obtained by a high‐temperature MD simulation. The Beeman algorithm is employed for the time propagation using a time‐step of 1.0 fs. The total simulation time of each of the independent MD simulations (NVE ensemble) was 4 ns. The used van‐der‐Waals cutoff is 12 Å. The default smooth particle mesh Ewald (SPME) approach^23^ as implemented in the Tinker code was used to describe the electrostatic charge‐charge interactions.

All DFT calculations were done using the Gaussian 16 code^24^ together with the B3LYP+GD3 functional^25^ and the cc‐pVDZ basis set^26^ for C,H,N and O and cc‐pVDZ‐PP basis set combined with the Stuttgart‐Dresden pseudopotentials for Zn.^27^

## Conflict of interest

The authors declare no conflict of interest.

## Supporting information

As a service to our authors and readers, this journal provides supporting information supplied by the authors. Such materials are peer reviewed and may be re‐organized for online delivery, but are not copy‐edited or typeset. Technical support issues arising from supporting information (other than missing files) should be addressed to the authors.

Supplementary
