# Supplementary material for: Atomistic Insight Into the Host–Guest Interaction of a Photoresponsive Metal–Organic Framework
Source: Chemistry. 2020 Jan 21;26(6):1263–8. doi: 10.1002/chem.201905139 (PMC7027908; doi:10.1002/chem.201905139)
Supplement: Supplementary file 1 — Supplementary [file CHEM-26-1263-s001.pdf]

CHEMISTRY
A European Journal

Supporting Information

Atomistic Insight Into the Host–Guest Interaction of a Photoresponsive Metal–Organic Framework

Elena Kolodzeiski[a, b] and Saeed Amirjalayer*[a, b]

chem_201905139_sm_miscellaneous_information.pdf

Atomistic Insight into the Host-Guest Interaction of a Photoresponsive Metal-Organic Framework

Elena Kolodzeiski[a], and Saeed Amirjalayer*[a]

[a] Physikalisches Institut, Westfälische Wilhelms-Universität Münster, Willhelm-Klemm-Strasse 10, 48149 Münster (Germany), Center for Nanotechnology (CeNTech) and Center for Multiscale Theory and Computation (CMTC) Heisenbergstrasse 11, 48149 Münster (Germany)

*e-mail: s.amirjalayer@wwu.de

Table of Contents

S1. Additional input structure for the GA S3

S2. Structural parameters of the reference system ***trans***$_A$ S3

S3. Structural parameters of the minima structures ***trans***$_B$, ***cis***$_A$ and ***cis***$_B$ S5

S4. Energy profile along the N-N-rotation S5

S5. Orientation profiles of molecules located in the α-pocket S6

S6. Free Energy topology of the guest molecules within the azo-MOF-5 S7

S7. Force field parameters S9

S8. Gaussian input files for geometry optimization and frequency calculation S13

S1. Additional input structure for the GA

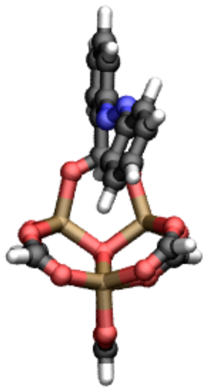

Figure S1: Ball-and stick model of ***trans**$_{A}$* with the C2-C3 dihedral angle rotated by 90° with respect to the optimized geometry.

S2. Structural parameters of the reference system *trans$_{A}$*

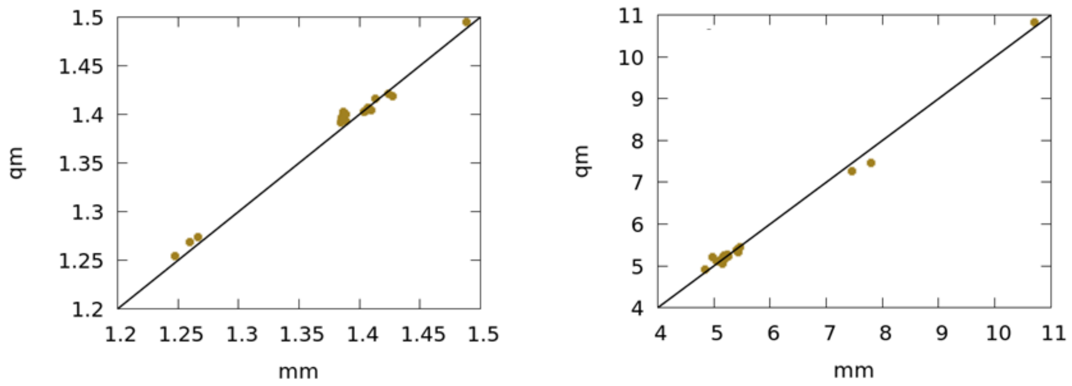

Figure S2: Comparison of the bond parameters of the optimized ***trans**$_{A}$* system including all C-atoms in the organic linker as well as the N-atoms and O1-atoms shown in Figure 1a). The data were calculated at the B3LYP+GD3 level (qm) and at the molecular mechanics (mm) level using our parametrized force field: bondlength of the optimized geometries (left, in Å) and the corresponding force constants (right, in mdyn/Å).

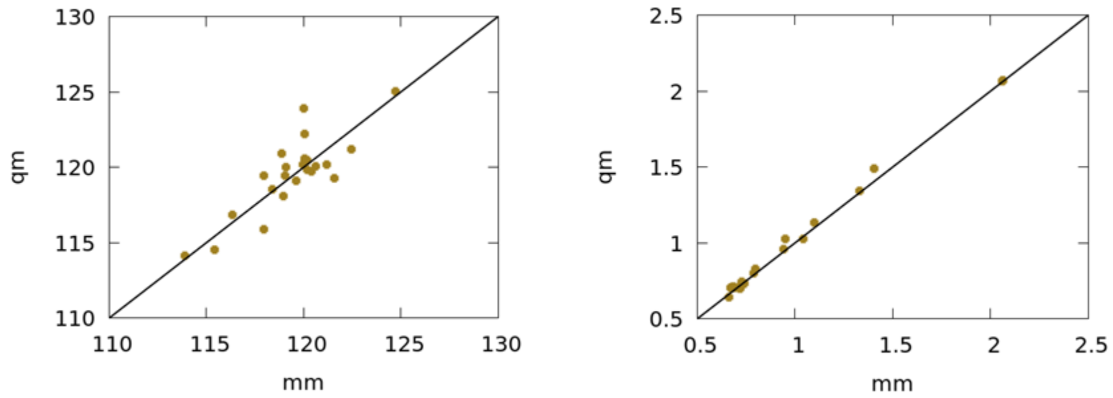

Figure S3: Comparison of the angle parameters of the optimized ***trans**$_{A}$* system including all C-atoms in the organic linker as well as the N-atoms and O1-atoms shown in Figure 1a). The data were calculated at the B3LYP+GD3 level (qm) and at the molecular mechanics (mm) level using our parametrized force field: angles of the optimized geometries (left, in degree) and the corresponding force constants (right, in mdynÅ /rad2).

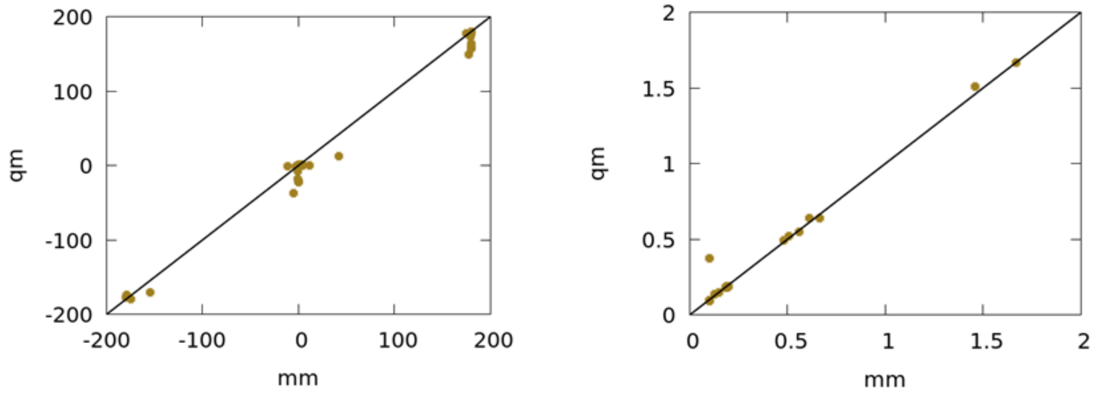

Figure S4: Comparison of the torsion parameters of the optimized ***trans***$_{A}$ system including all C-atoms contained in the organic linker as well as the N-atoms and O1-atoms shown in Figure 1a). The data were calculated at the B3LYP+GD3 level (qm) and at the molecular mechanics (mm) level using our parametrized force field: dihedral angles of the optimized geometries (left, in degree) and the corresponding force constants (right, in kcal/mol).

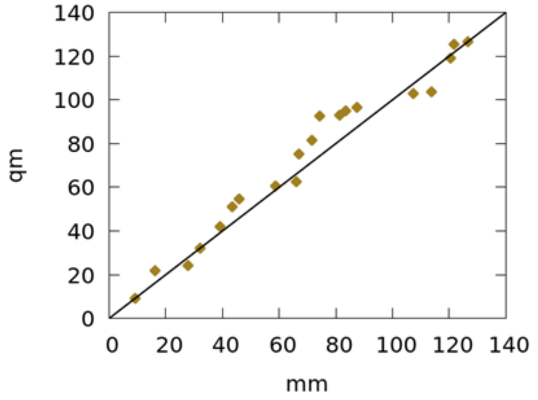

Figure S5: Comparison of the normal modes (in 1/cm) of the reference system ***trans***$_{A}$ calculated on QM and MM level.

S3. Structural parameters of the minima structures ***trans*$_B$**, ***cis*$_A$** and ***cis*$_B$**

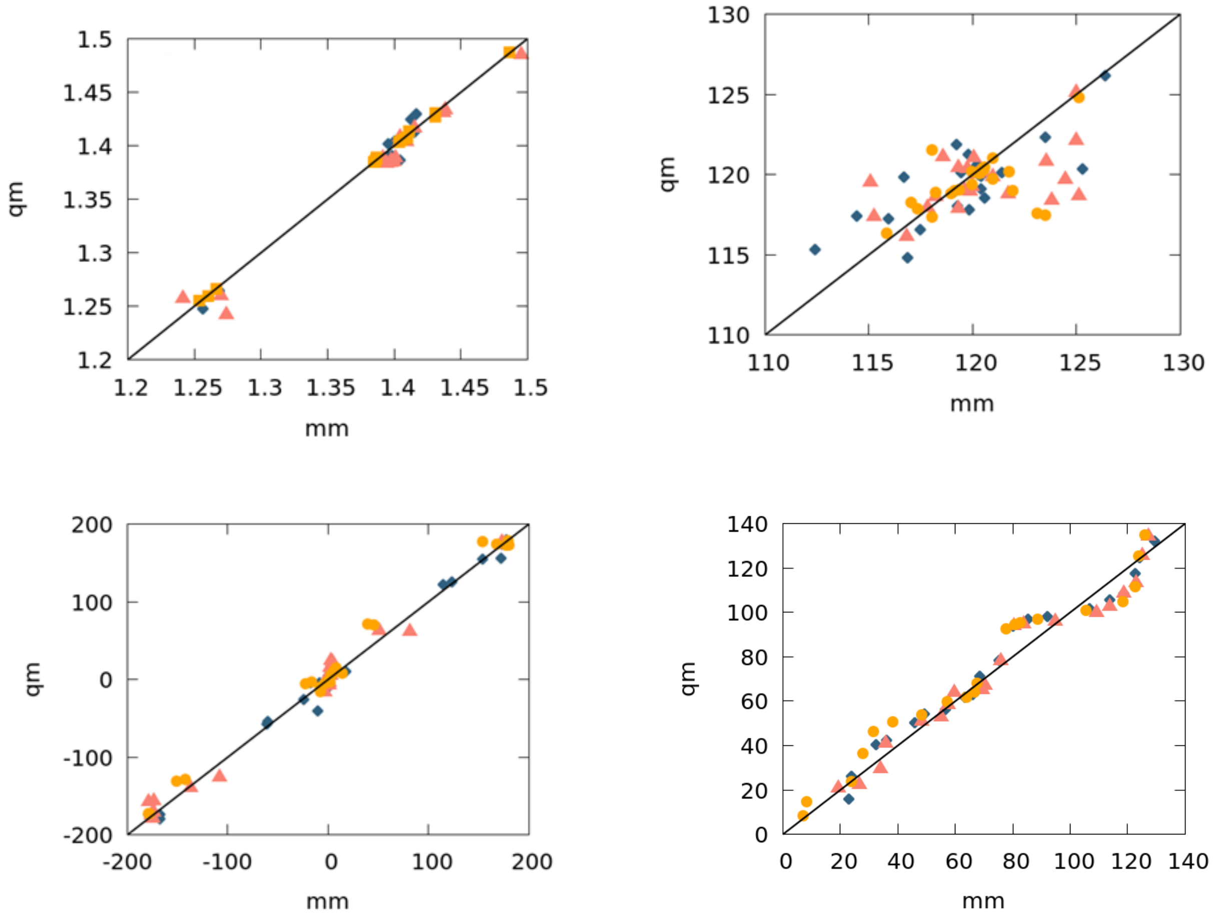

Figure S6: Bond length in Å (top left), angles in degree (top right), dihedral angles in degree (bottom left) and normal modes in 1/cm (bottom right) of the ***trans*$_B$** (blue diamonds), ***cis*$_A$** (yellow squares), ***cis*$_B$** (pink triangles) calculated on QM and MM level. Considered are all C and N-atoms corresponding to the organic linker and the O1 atoms from Figure 1a).

S4. Energy profile along the N-N-rotation

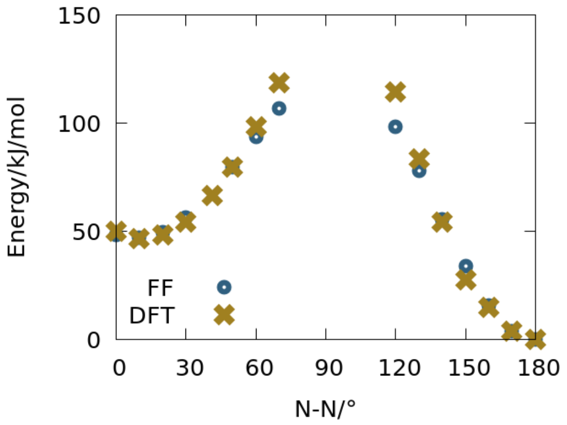

Figure S7: Energy profile along the rotation of the dihedral angle N6-N7 (Figure 1a). The local minimum around 10° corresponds to ***cis*$_A$** and the absolute minimum around 180° corresponds to ***trans*$_A$**. The blue circles denote the data obtained by the parametrized force field and the green crosses the corresponding calculated energies at the B3LYP+GD3 level.

S5. Orientation profiles of molecules located in the α-pocket

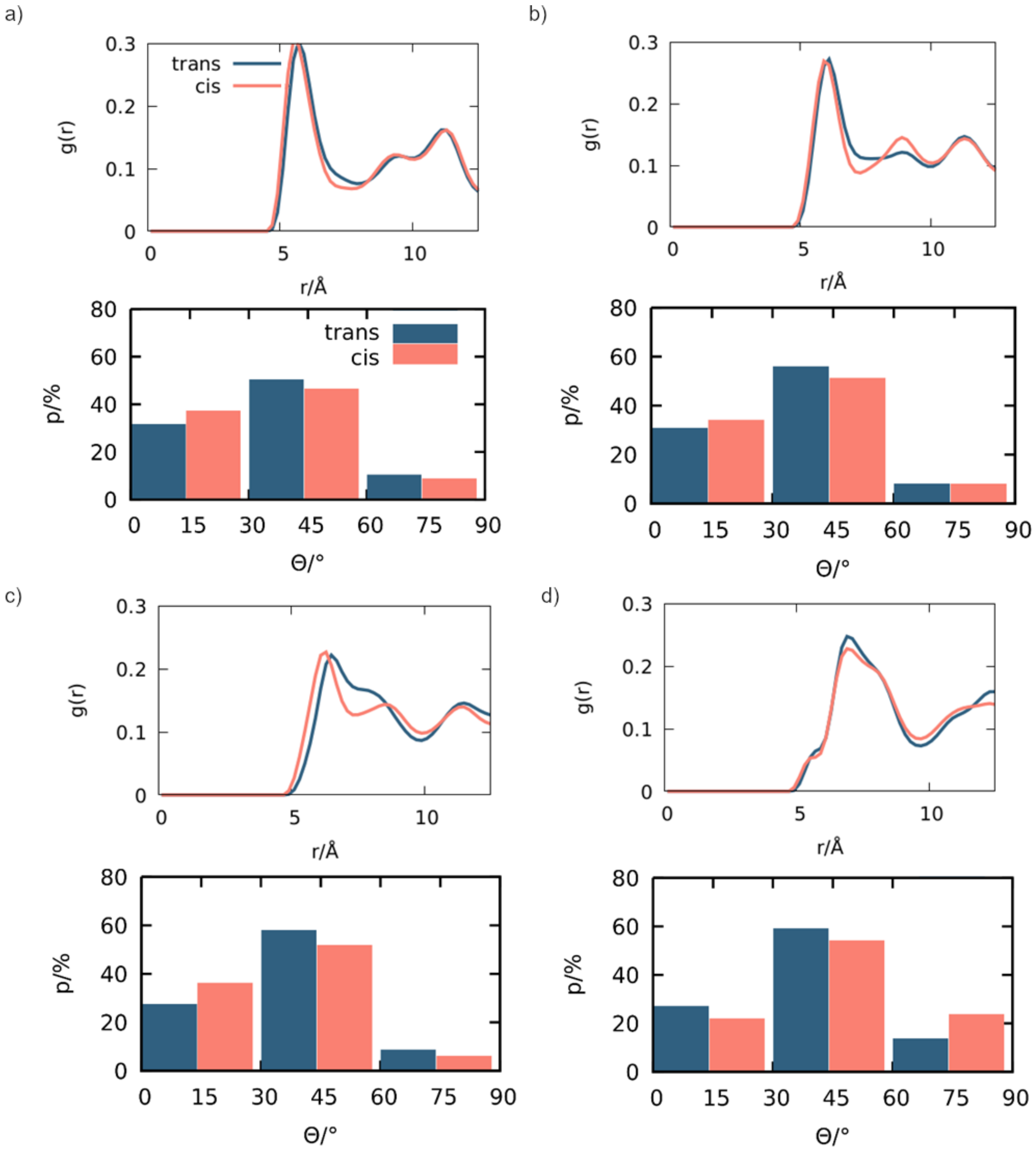

Figure S8: Radial distribution functions (RDF) between the central O-atoms of the Zn4O corners and the center of mass of a) benzene, b) toluene, c) m-xylene, d) mesitylene molecules (top) and the corresponding probability distribution of the orientation of the molecules in the α-pocket defined by the distribution of the θ-angle (bottom). The first peak in the RDF is used for the analysis of the orientation.

S6. Free Energy topology of the guest molecules within the azo-MOF-5

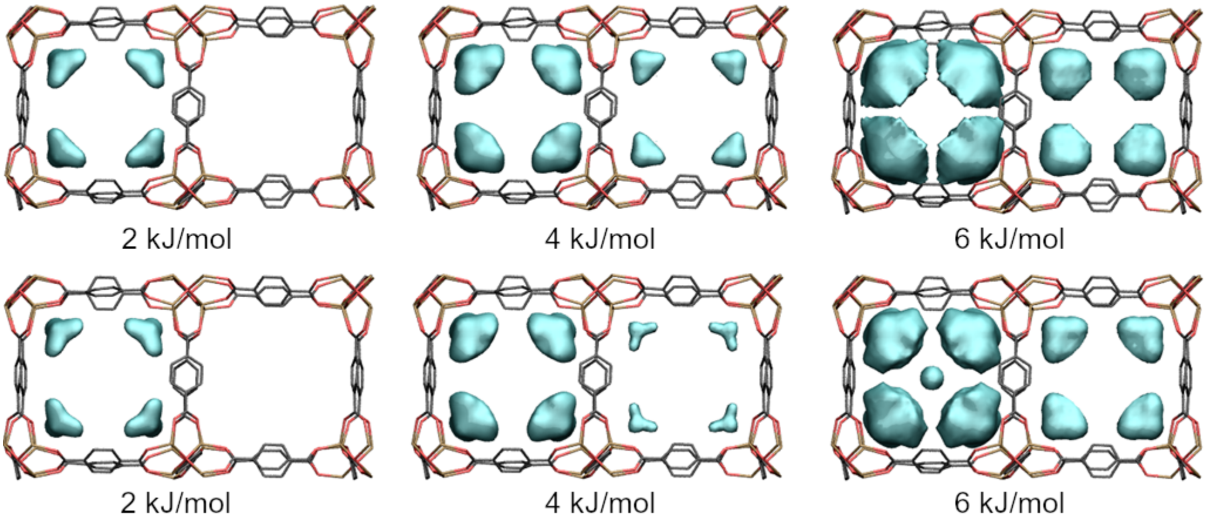

Figure S9: Free energy topology of benzene guest molecules in azo-MOF-5: ***all-trans*** azo-MOF (top) and ***all-cis*** azo-MOF (bottom) for different ΔG-isovalues.

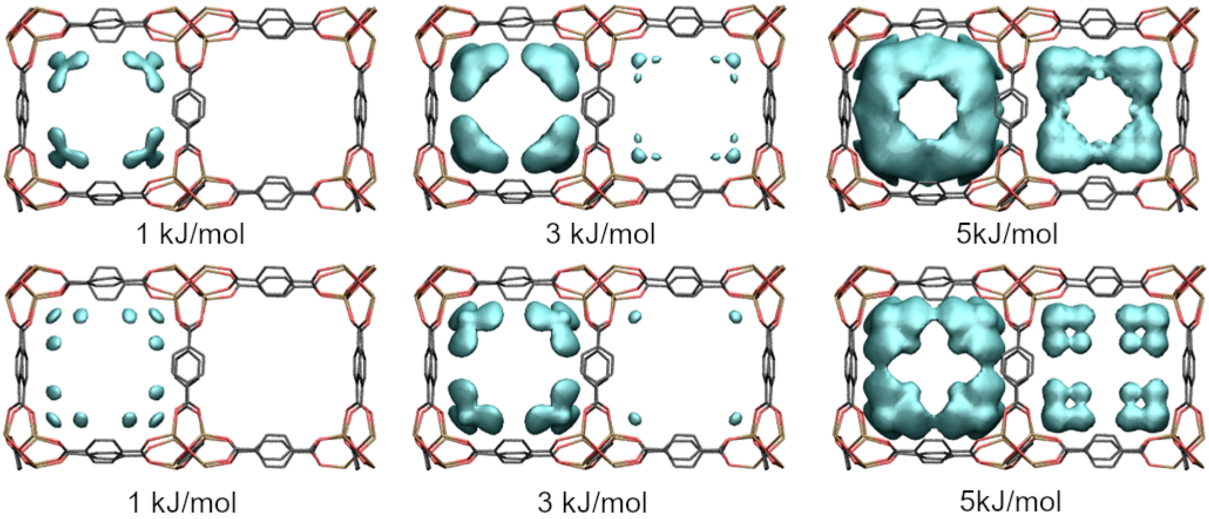

Figure S10: Free energy topology of toluene guest molecules in azo-MOF-5: ***all-trans*** azo-MOF (top) and ***all-cis*** azo-MOF (bottom) for different ΔG-isovalues.

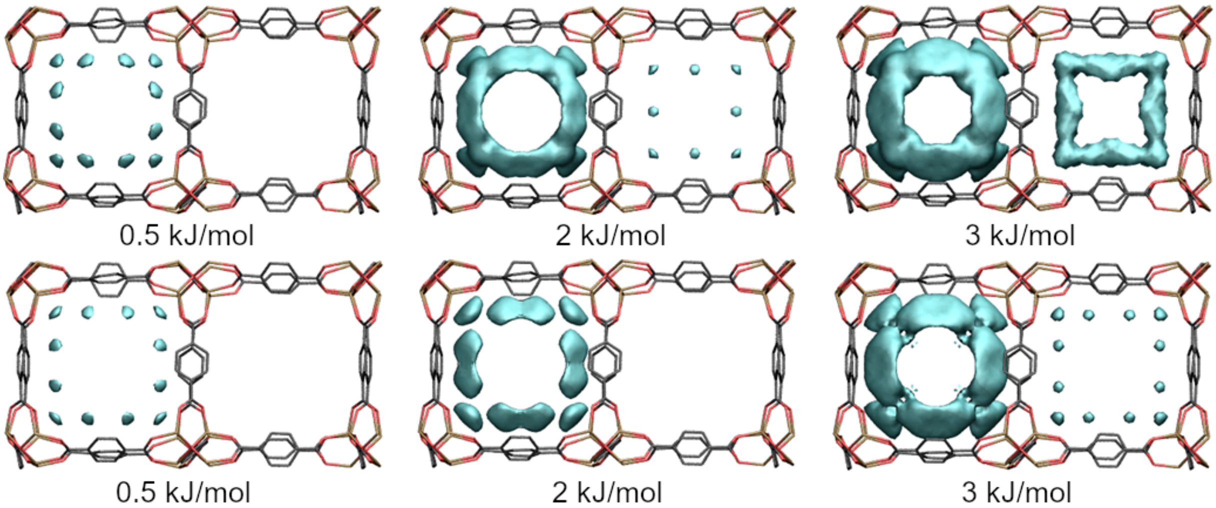

Figure S11: Free energy topology of m-xylene guest molecules in azo-MOF-5: ***all-trans*** azo-MOF (top) and ***all-cis*** azo-MOF (bottom) for different ΔG-isovalues.

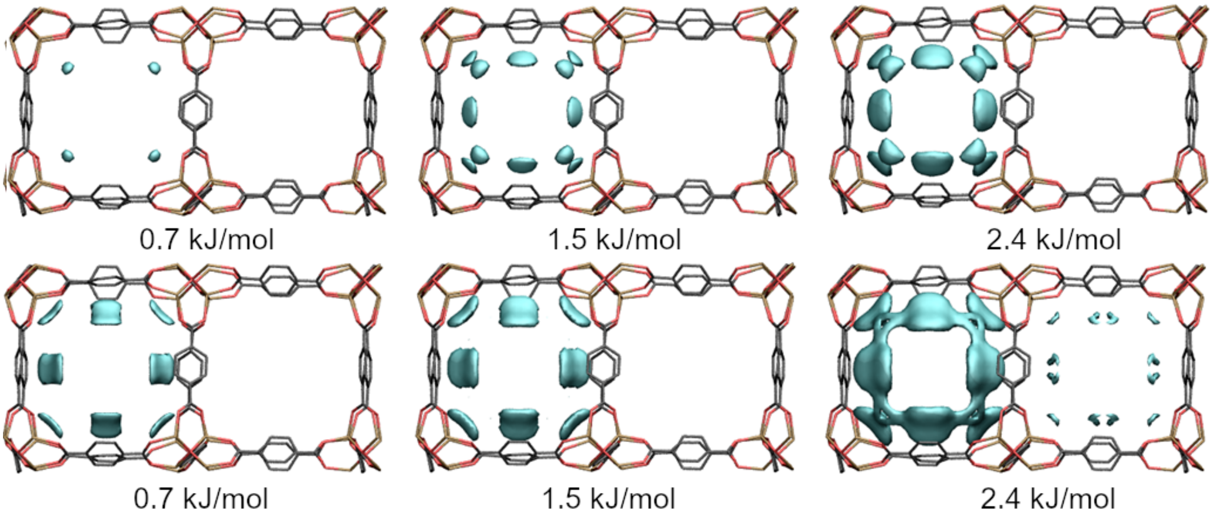

Figure S12: Free energy topology of mesitylene guest molecules in azo-MOF-5: ***all-trans*** azo-MOF (top) and ***all-cis*** azo-MOF (bottom) for different ΔG-isovalues.

S7. Force field parameters

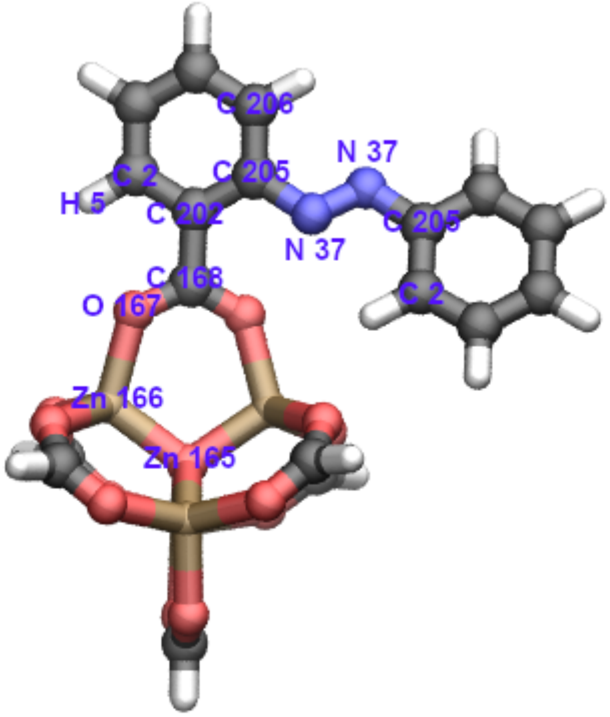

Figure S13: Graphic of the building block ***trans$_A$*** to define the atom-types used in the force field.

Table S1: Parameters for the bond stretch terms for the extended force field

Bond stretch		*kb*/mdyn/Å	r_b^{ref}/Å
2	2	7.346	1.380
206	5	5.150	1.101
2	37	5.413	1.434
168	169	5.140	1.103
205	37	5.414	1.416
202	2	6.636	1.400
2	205	6.670	1.400
206	205	6.670	1.400
206	2	7.346	1.380
37	37	10.857	1.245
202	205	6.461	1.400
202	168	5.238	1.467
202	206	6.636	1.400

Table S2: Parameters for the in-plane angle bending terms for the extended force field

**In-plane angle bending**			ka/mdyn Å /rad^2	Θ_a^{ref}/deg
206	2	2	0.760	122.000
2	206	5	0.490	120.000
206	2	5	0.490	120.000
2	2	37	0.612	124.682
2	2	168	0.723	115.312
2	37	37	1.748	113.609
2	202	168	0.893	115.312
2	205	2	0.760	122.000
165	166	167	0.263	105.600
166	165	166	0.026	109.637
166	167	168	0.164	135.340
167	168	167	1.160	128.276
167	166	167	0.263	105.600
167	168	202	1.140	117.523
202	2	2	0.992	122.000
202	205	2	0.870	120.000
202	205	206	0.600	120.000
205	2	2	0.766	108.600
205	206	2	1.042	108.600
205	2	5	0.490	120.000
205	206	5	0.490	120.000
205	2	168	0.723	115.312
206	205	37	1.033	115.694
2	205	37	1.097	115.694
205	37	37	1.904	113.217
202	205	37	2.343	116.085
205	202	2	0.555	129.066
205	202	168	1.838	115.002
205	206	202	0.760	122.000
202	206	5	0.490	120.000
202	206	2	0.760	122.000
206	205	2	0.760	122.000
206	202	168	0.723	115.312
206	202	2	0.760	122.000
167	168	169	0.630	116.280

Table S3: Parameters for the torsion terms for the extended force field. The parameter V_t^2 are in kcal/mol, τ^2_t in degree and *n* defines the order of the fourier-term.

**torsion**				V_t^2	τ^2_t	*n*	V_t^2	τ^2_t	*n*	V_t^2	τ^2_t	*n*	V_t^2	τ^2_t	*n*
2	2	2	206	0.148	0	1	3.973	180	2	0.000	0	3	0.000	0	4
2	2	206	5	0.000	0	1	5.385	180	2	0.000	0	3	0.000	0	4
206	2	2	5	0.000	0	1	5.385	180	2	0.000	0	3	0.000	0	4
2	2	202	205	2.306	0	1	5.600	180	2	0.000	0	3	0.000	0	4
2	2	205	37	0.190	0	1	15.844	180	2	0.000	0	3	0.000	0	4
2	206	205	37	0.867	0	1	7.443	180	2	0.000	0	3	0.000	0	4
2	2	205	202	44.880	0	1	0.003	180	2	0.000	0	3	0.000	0	4
2	206	205	202	1.242	0	1	3.284	180	2	0.000	0	3	0.000	0	4
2	202	205	37	7.282	0	1	19.216	180	2	0.000	0	3	0.000	0	4
2	205	2	2	10.052	0	1	2.978	180	2	0.000	0	3	0.000	0	4
2	205	2	5	0.000	0	1	5.385	180	2	0.000	0	3	0.000	0	4
2	205	2	168	0.000	0	1	5.983	180	2	0.000	0	3	0.000	0	4
206	205	37	37	22.666	0	1	0.338	180	2	0.466	0	3	0.000	0	4
2	205	37	37	25.359	0	1	2.489	180	2	1.778	0	3	0.000	0	4
206	205	202	2	0.500	0	1	3.800	180	2	0.000	0	3	0.000	0	4
2	205	202	2	0.000	0	1	5.983	180	2	0.000	0	3	0.000	0	4
2	205	202	168	0.000	0	1	5.983	180	2	0.000	0	3	0.000	0	4
206	205	202	168	0.000	0	1	5.983	180	2	0.000	0	3	0.000	0	4
5	2	206	5	0.000	0	1	6.881	180	2	0.000	0	3	0.000	0	4
5	2	205	5	0.000	0	1	6.881	180	2	0.000	0	3	0.000	0	4
5	2	205	37	0.000	0	1	0.000	180	2	0.000	0	3	0.000	0	4
5	206	205	37	0.000	0	1	0.000	180	2	0.000	0	3	0.000	0	4
166	167	168	167	0.000	0	1	4.108	180	2	0.000	0	3	0.000	0	4
166	167	168	169	0.779	0	1	2.694	180	2	0.610	0	3	0.000	0	4
166	167	168	202	0.113	0	1	3.138	180	2	3.688	0	3	0.000	0	4
167	168	202	205	0.000	0	1	1.389	180	2	0.000	0	3	0.013	180	4
167	168	202	2	0.000	0	1	4.690	180	2	0.000	0	3	0.198	180	4
168	202	205	37	0.472	0	1	8.757	180	2	0.000	0	3	0.000	0	4
202	205	37	37	13.156	0	1	8.528	180	2	1.442	0	3	0.000	0	4
202	205	2	5	0.000	0	1	5.385	180	2	0.000	0	3	0.000	0	4
202	205	206	5	0.000	0	1	5.385	180	2	0.000	0	3	0.000	0	4
205	206	2	2	16.480	0	1	5.114	180	2	0.000	0	3	0.000	0	4
205	2	2	2	0.000	0	1	13.000	180	2	0.000	0	3	0.000	0	4
205	2	2	5	0.000	0	1	5.385	180	2	0.000	0	3	0.000	0	4
205	206	2	5	0.000	0	1	5.385	180	2	0.000	0	3	0.000	0	4
202	2	2	2	0.382	0	1	9.510	180	2	0.000	0	3	0.000	0	4
205	37	37	205	0.020	0	1	27.186	180	2	2.709	0	3	0.000	0	4
205	202	2	5	0.000	0	1	5.385	180	2	0.000	0	3	0.000	0	4
202	206	205	2	0.000	0	1	5.983	180	2	0.000	0	3	0.000	0	4
205	206	202	2	0.000	0	1	5.983	180	2	0.000	0	3	0.000	0	4
205	206	202	168	0.000	0	1	5.983	180	2	0.000	0	3	0.000	0	4
168	202	206	5	0.000	0	1	5.983	180	2	0.000	0	3	0.000	0	4
206	205	2	2	0.000	0	1	5.983	180	2	0.000	0	3	0.000	0	4
2	2	202	206	0.000	0	1	5.983	180	2	0.000	0	3	0.000	0	4
5	206	205	2	0.000	0	1	5.385	180	2	0.000	0	3	0.000	0	4

5	206	202	2	0.000	0	1	5.385	180	2	0.000	0	3	0.000	0	4
206	205	2	5	0.000	0	1	5.385	180	2	0.000	0	3	0.000	0	4
5	2	202	206	0.000	0	1	5.385	180	2	0.000	0	3	0.000	0	4
202	2	2	202	0.382	0	1	9.510	180	2	0.000	0	3	0.000	0	4
202	206	205	37	0.867	0	1	7.443	180	2	0.000	0	3	0.000	0	4
202	206	205	202	1.242	0	1	3.284	180	2	0.000	0	3	0.000	0	4
167	168	202	206	0.000	0	1	2.122	180	2	0.000	0	3	0.000	0	4

Table S4: Parameters for the combined stretch-stretch and stretch-bend cross terms for the extended force field.

**Combined stretch-stretch and stretch-bend cross terms**			k_{sb1}/mdyn/rad	k_{s2}/mdyn/rad	k_{ss}/mdyn/Å
167	168	169	0.5	0.2	0.3

Table S5: Parameters for the Out-of-plane terms of the extended force field

**Out-of-plane bending**		Θ_0^{ref}/deg	k_0/mdynÅ/rad^2
2	206	0.0	0.200
206	2	0.0	0.200
2	205	0.0	0.754
37	37	0.0	0.150
168	169	0.0	1.313
205	37	0.0	0.078
205	2	0.0	0.481
205	206	0.0	0.481
206	205	0.0	0.754
37	205	0.0	0.043
206	5	0.0	0.200
202	2	0.0	0.508
202	168	0.0	0.564
202	205	0.0	0.539
2	202	0.0	0.601
205	202	0.0	0.800
168	202	0.0	0.649
206	202	0.0	0.200
202	206	0.0	0.200

Table S6: Parameters for the non-bonded terms of the extended force field

Atom type	radius 0.5 d_n^0/Å	ε_n/kcal/mol	bondlength reduction factor	charge
205	1.96	0.056		0.21
37	1.93	0.043		-0.21
169	1.62	0.020	0.923	0.02
206	1.96	0.056		0.12

S8. Gaussian input files for geometry optimization and frequency calculations

Table S8: Example file for the geometry optimization calculations

```
%chk=example.chk
#P B3LYP/gen pseudo=read opt=tight EmpiricalDispersion=GD3

title

0 1
c -5.278693 3.219826 0.322841
c -4.216125 2.325971 0.109525
c -2.910377 2.854307 0.041994
c -2.672565 4.236834 0.036371
c -3.754663 5.092416 0.172143
c -5.038895 4.587103 0.326950
n -4.496629 0.927273 0.050442
n -3.671400 0.168070 -0.495850
c -3.915081 -1.233426 -0.534474
c -4.961092 -1.820639 0.198637
c -5.127009 -3.194889 0.145055
c -4.265023 -3.975628 -0.610515
c -3.224157 -3.397638 -1.321146
c -3.035058 -2.026607 -1.285537
c -1.730273 1.966426 0.052116
o -1.589585 1.229271 1.069395
zn -0.239838 -0.113879 1.562456
o 1.124534 -0.261282 0.144542
zn 1.105733 -2.098654 -0.568791
o 2.872528 -2.959871 -0.362026
c 3.932450 -2.523823 0.165916
o 4.125801 -1.392671 0.690581
zn 2.901676 0.146101 0.892872
o 2.792919 0.594068 2.814257
c 1.785968 0.661048 3.571798
o 0.580062 0.438427 3.275213
o -0.911584 2.094912 -0.902204
zn 0.700923 1.025979 -1.292052
o 0.412497 0.084863 -3.007243
c 0.431731 -1.146729 -3.279986
o 0.655660 -2.108785 -2.494513
o 2.187908 2.299326 -1.567753
c 3.295876 2.398816 -0.972385
o 3.733548 1.683129 -0.029883
o -1.142457 -1.842427 1.880982
c -1.011288 -2.951098 1.293739
o -0.215363 -3.239181 0.357172
h -3.590528 6.183295 0.179356
h -1.646001 4.630762 -0.044177
h -6.305148 2.836213 0.443036
h 3.961535 3.208278 -1.317079
h 4.793005 -3.214167 0.169134
h -1.671192 -3.765067 1.638660
h 0.227891 -1.414988 -4.330491
h 1.983731 0.948620 4.618376
h -5.883836 5.283831 0.457966
h -5.645389 -1.203630 0.803478
h -5.947295 -3.669335 0.709060
h -4.406793 -5.069175 -0.644187
h -2.541811 -4.029828 -1.914017
h -2.215362 -1.560830 -1.855326

H     0
S   3   1.00
     13.0100000              0.0196850
      1.9620000              0.1379770
      0.4446000              0.4781480
S   1   1.00
      0.1220000              1.0000000
P   1   1.00
      0.7270000              1.0000000
```

```
****
C     0
S   8   1.00
   6665.0000000              0.0006920
   1000.0000000              0.0053290
    228.0000000              0.0270770
     64.7100000              0.1017180
     21.0600000              0.2747400
      7.4950000              0.4485640
      2.7970000              0.2850740
      0.5215000              0.0152040
S   8   1.00
   6665.0000000             -0.0001460
   1000.0000000             -0.0011540
    228.0000000             -0.0057250
     64.7100000             -0.0233120
     21.0600000             -0.0639550
      7.4950000             -0.1499810
      2.7970000             -0.1272620
      0.5215000              0.5445290
S   1   1.00
      0.1596000              1.0000000
P   3   1.00
      9.4390000              0.0381090
      2.0020000              0.2094800
      0.5456000              0.5085570
P   1   1.00
      0.1517000              1.0000000
D   1   1.00
      0.5500000              1.0000000
****
N     0
S   8   1.00
   9046.0000000              0.0007000
   1357.0000000              0.0053890
    309.3000000              0.0274060
     87.7300000              0.1032070
     28.5600000              0.2787230
     10.2100000              0.4485400
      3.8380000              0.2782380
      0.7466000              0.0154400
S   8   1.00
   9046.0000000             -0.0001530
   1357.0000000             -0.0012080
    309.3000000             -0.0059920
     87.7300000             -0.0245440
     28.5600000             -0.0674590
     10.2100000             -0.1580780
      3.8380000             -0.1218310
      0.7466000              0.5490030
S   1   1.00
      0.2248000              1.0000000
P   3   1.00
     13.5500000              0.0399190
      2.9170000              0.2171690
      0.7973000              0.5103190
P   1   1.00
      0.2185000              1.0000000
D   1   1.00
      0.8170000              1.0000000
****
O     0
S   8   1.00
  11720.0000000              0.0007100
   1759.0000000              0.0054700
    400.8000000              0.0278370
    113.7000000              0.1048000
     37.0300000              0.2830620
     13.2700000              0.4487190
      5.0250000              0.2709520
      1.0130000              0.0154580
S   8   1.00
```

```
 11720.0000000         -0.0001600
  1759.0000000         -0.0012630
   400.8000000         -0.0062670
   113.7000000         -0.0257160
    37.0300000         -0.0709240
    13.2700000         -0.1654110
     5.0250000         -0.1169550
     1.0130000          0.5573680
S   1   1.00
     0.3023000          1.0000000
P   3   1.00
    17.7000000          0.0430180
     3.8540000          0.2289130
     1.0460000          0.5087280
P   1   1.00
     0.2753000          1.0000000
D   1   1.00
     1.1850000          1.0000000
****
Zn     0
S   7   1.00
   629.1260000          0.0005590
    62.9635000         -0.0096800
    39.5791000          0.0623540
    11.9173000         -0.4148790
     2.5082400          0.7544660
     1.0301900          0.4589960
     0.1540020          0.0116640
S   7   1.00
   629.1260000         -0.0001210
    62.9635000          0.0014890
    39.5791000         -0.0127370
    11.9173000          0.0972460
     2.5082400         -0.2199170
     1.0301900         -0.2364740
     0.1540020          0.5812910
S   7   1.00
   629.1260000          0.0004210
    62.9635000         -0.0051090
    39.5791000          0.0414470
    11.9173000         -0.3416430
     2.5082400          1.4386170
     1.0301900         -0.9824290
     0.1540020         -1.1876310
S   1   1.00
     0.0527950          1.0000000
P   6   1.00
    92.9034000          0.0024880
    19.7452000         -0.0791360
     4.5507300          0.3880590
     2.0019000          0.5093540
     0.8438790          0.2245750
     0.2033810          0.0126800
P   6   1.00
    92.9034000         -0.0004030
    19.7452000          0.0154470
     4.5507300         -0.0843250
     2.0019000         -0.1364890
     0.8438790          0.0008020
     0.2033810          0.5157920
P   6   1.00
    92.9034000         -0.0005300
    19.7452000          0.0247430
     4.5507300         -0.1424780
     2.0019000         -0.2340320
     0.8438790          0.0921870
     0.2033810          0.8377700
P   1   1.00
     0.0595720          1.0000000
D   5   1.00
    71.2766000          0.0158950
    22.7604000          0.0924540
```

```
      8.3236900             0.2594720
      3.1687500             0.4033140
      1.1470300             0.3878340
D   5   1.00
     71.2766000             -0.0225160
     22.7604000             -0.1333030
      8.3236900            -0.3932530
      3.1687500            -0.3512140
      1.1470300             0.4061710
D   1   1.00
      0.3666430             1.0000000
F   1   1.00
      3.2168000             1.0000000
****

ZN     0
ZN-ECP     4     10
g-ul potential
  1
2      1.0000000              0.0000000
s-ul potential
  2
2     34.1740010            399.9863990
2     14.4563710             85.4897500
p-ul potential
  4
2     39.8886830             92.3810770
2     39.6550170            184.7711760
2     15.2905460             23.0025410
2     14.9035240             46.0574270
d-ul potential
  4
2     43.7082960            -13.6907340
2     43.6985360            -20.5439800
2     15.1507180             -1.3161540
2     15.2824410             -1.8387150
f-ul potential
  2
2      8.1600140            -0.3703600
2     12.2284220             -1.0629430
```

Table S9: Example file for the subsequent frequency calculation based on the performed geometry optimization

```
%chk=example.chk
#P B3LYP/chkbas geom=allcheck EmpiricalDispersion=GD3 guess=read freq
```
